# Catalyzing plant science research with RNA-seq

**DOI:** 10.3389/fpls.2013.00066

**Published:** 2013-04-01

**Authors:** Laetitia B. B. Martin, Zhangjun Fei, James J. Giovannoni, Jocelyn K. C. Rose

**Affiliations:** ^1^Department of Plant Biology, Cornell UniversityIthaca, NY, USA; ^2^Boyce Thompson Institute for Plant ResearchIthaca, NY, USA; ^3^Robert W. Holly Center for Agriculture and Health, United States Department of Agriculture-Agricultural Research ServiceIthaca, NY, USA

**Keywords:** RNA-seq, plant transcriptome, transcriptomics, systems biology, next generation sequencing

## Abstract

Next generation DNA sequencing technologies are driving increasingly rapid, affordable and high resolution analyses of plant transcriptomes through sequencing of their associated cDNA (complementary DNA) populations; an analytical platform commonly referred to as RNA-sequencing (RNA-seq). Since entering the arena of whole genome profiling technologies only a few years ago, RNA-seq has proven itself to be a powerful tool with a remarkably diverse range of applications, from detailed studies of biological processes at the cell type-specific level, to providing insights into fundamental questions in plant biology on an evolutionary time scale. Applications include generating genomic data for heretofore unsequenced species, thus expanding the boundaries of what had been considered “model organisms,” elucidating structural and regulatory gene networks, revealing how plants respond to developmental cues and their environment, allowing a better understanding of the relationships between genes and their products, and uniting the “omics” fields of transcriptomics, proteomics, and metabolomics into a now common systems biology paradigm. We provide an overview of the breadth of such studies and summarize the range of RNA-seq protocols that have been developed to address questions spanning cell type-specific-based transcriptomics, transcript secondary structure and gene mapping.

## INTRODUCTION

Next generation sequencing (NGS) is underpinning an ongoing revolution in the life sciences and it is now difficult to identify areas of biology that are not already being profoundly affected by the massive amounts of high quality DNA sequence information that has been generated cost-effectively and efficiently, thanks to the rapid advancement of sequencing technologies. Plant biology is naturally no exception to this revolution; indeed the ease of genetic analyses in many plant species and the value of crop species have made plant science an especially fertile area for many of the “omics” technologies. Plant scientists are rapidly moving on from a decade where the first genome sequence of a plant, that of *Arabidopsis thaliana *(The Arabidopsis Genome Initiative, 2000), provided the major impetus for monumental forays into plant molecular investigations, to the present day where the growing number of sequenced plant genomes^1^ is driving biological and evolutionary discovery across the plant taxonomic range.

In parallel with this explosion of genome sequence information, NGS has changed the scope and scale of transcriptome analysis and gene expression studies. RNA-sequencing (RNA-seq) technologies, which apply the principles of NGS to the complementary DNAs (cDNAs) derived from transcript populations, were first used to study plants only a few years ago (Weber et al., 2007) and now provide ready access to high resolution transcriptome information to an extent that was once unimaginable. This is exemplified by the 1KP project^2^, which aims to sequence the transcriptomes of 1,000 plant species, and is just one of many current initiatives that are radically expanding the breadth and depth of our understanding of plant gene expression and evolution. Due to its accuracy and the ease of meaningful comparisons of samples not necessarily generated together, or even as part of the same experiment, RNA-seq is replacing other methods of quantifying transcript expression, including cDNA- and expressed sequenced tag (EST)-based microarray platforms (Alba et al., 2004), as it overcomes many of their limitations (for an overview of RNA-seq technologies and comparisons with previous transcript detection technologies, see Wang et al., 2009). For example, RNA-seq approaches have an open architecture, meaning that they are not restricted to detecting only those transcripts that are represented on microarrays, and also exhibit more extreme upper and lower limits of detection, which allows more accurate quantification of differential transcript expression, as well as the identification of low-abundance transcripts. Furthermore, no previous genome sequence knowledge is necessary, as RNA-seq data sets themselves can be used to create sequence assemblies for subsequent mapping of RNA-seq reads, along with the potential for detecting exon/exon boundaries, alternative splicing and novel transcribed regions in a single sequencing run. However, despite these advantages, RNA-seq profiling platforms come with their own practical challenges. Existing RNA-seq techniques generate large numbers of relatively short reads for a particular transcript and so the accurate assembly and annotation of the huge amounts of data generated by each run is still computationally difficult (Schliesky et al., 2012). Moreover, various biases can be introduced during the RNA fragmentation step prior to library construction, and cDNA fragmentation enriches the reads mapping the 3′ end of transcripts (Wang et al., 2009).

Nonetheless, RNA-seq has emerged as a remarkable enabling technology that is increasingly being adopted by plant researchers from a broad range of disciplines and examples of some of the associated applications and fields of research are presented in this review.

## IMPROVING GENOME ANNOTATION WITH TRANSCRIPTOMIC DATA

More than a decade after the publication of the first draft of the *A. thaliana* genome sequence (The Arabidopsis Genome Initiative, 2000) its annotation continues to be improved. Large amounts of Sanger sequencing-generated EST data provided the initial basis for gene identification and expression profiling (Zhu et al., 2003), but such data are expensive and time consuming to generate, are inherently biased against low-abundance transcripts and are typically enriched in transcript termini (Filichkin et al., 2010). RNA-seq circumvents these limitations and provides accurate resolution of splice junctions and alternative splicing events. For example, a survey of the *Arabidopsis* transcriptome using single-base resolution Illumina-generated reads identified thousands of novel alternatively spliced transcripts and indicated that at least 42% of intron-containing genes are alternatively spliced (Filichkin et al., 2010). This percentage is considerably higher than previous estimations and is even greater (61%) when only the multiexonic genes are sampled (Marquez et al., 2012). Similarly, approximately 48% of rice (*Oryza sativa*) genes show alternative splicing patterns (Lu et al., 2010), although more species need to be analyzed to determine whether this proportion is common. Mining RNA-seq data in search of transcription start site (TSS) variation is also improving gene structure annotation and alternative TSSs have been detected in ~10,000 loci through analyses of full-length *Arabidopsis* and rice cDNAs (Tanaka et al., 2009). RNA-seq analysis also helps elucidate full-length transcript sequences, as has been demonstrated in a study where ~10% of the untranslated region (UTR) boundaries of rice genes could be extended (Lu et al., 2010).

An ideal genome annotation would identify both genes that show invariant transcript sequences and those that exhibit alternative splicing, and additionally link these events to specific spatial, temporal, developmental, and/or environmental cues. Efforts in this direction are already underway and, as an example, it has been reported that abiotic stress in *Arabidopsis* can increase or decrease the proportions of apparently unproductive isoforms for some key regulatory genes, supporting the hypothesis that alternative splicing is an important mechanism in the regulation of gene function (Filichkin et al., 2010).

For many heterozygous and out-crossing species, genome sequencing and annotation can only be considered complete once the breadth of intra-species polymorphism is also considered. The high quality reference genome of *A. thaliana* is based on the ecotype Columbia (Col-0). It has been reported that polymorphisms between different *A. thaliana *accessions is relatively high, with one single nucleotide polymorphism (SNP) every ~200 bp (Ossowski et al., 2008). The complete re-sequencing of the transcriptomes and annotation of different accessions may thus help interpret the functional consequences of polymorphism (Gan et al., 2011). To this end, utilizing genomic and transcriptomic data for *in silico* gene prediction results in a more reliable annotated genome, with information on SNPs, insertion/deletions (indels), splice variants and expression variation. Furthermore, with its greater sensitivity, RNA-seq enables the detection of antisense transcripts and transcribed intergenic regions; topics that are discussed further in Section “Identifying and Characterizing Novel Non-Coding RNAs.”

## GENERATING GENOMIC AND ENABLING PROTEOMIC RESOURCES FOR “NON-MODEL” SPECIES

Despite the recent upsurge in published plant genome sequences, they still represent a very small fraction of plant taxonomic diversity and the availability of transcriptomic information based on Sanger sequence-derived ESTs is similarly sparse, rendering the study of “non-model” species challenging. The very large genomes often encountered in plants, frequently associated with high sequence repeat regions, makes *de novo* sequencing of the transcriptome an attractive alternative to generate genetic resources for species that are of considerable biological interest for reasons that relate to factors such as their evolutionary significance or economic importance. Examples of recent such initiatives include fern (Der et al., 2011), eucalyptus (Mizrachi et al., 2010), garlic (Sun et al., 2012), pea (Franssen et al., 2011), chestnut (Barakat et al., 2009), chickpea (Garg et al., 2011), olive (Alagna et al., 2009), safflower (Lulin et al., 2012), and Japanese knotweed (Hao et al., 2011). The annotation of genes identified by *de novo* sequencing typically relies on identifying homologs, and ideally orthologs, in species with an annotated genome if no appropriate EST databases are available. An example of such annotation, using a pre-existing EST database associated with the species of interest, was reported for melon (Dai et al., 2011). Use of the annotated genome of a close-related species (e.g., Barrero et al., 2011) is preferable, but if none is available, the *A. thaliana *genome sequence is still widely regarded as the “gold standard” and can be extremely valuable to this end (e.g., Bräutigam et al., 2011). Further confirmation can then be sought by interrogating additional plant databases (e.g., Dassanayake et al., 2009; Edwards et al., 2012), although this depends on the standard of annotation and care should be taken that the database of interest is of high quality.

*De novo *RNA-seq to identify genetic polymorphisms also has great potential as a platform for molecular breeding, wherein multiple cultivars or close-related species with variations in traits of interest are sequenced and genetic variation is identified. This then allows the generation of molecular markers to facilitate progeny selection and molecular genetics research. As an example of this approach, the identification of 12,000 single sequence repeats (SSRs) in a single RNA-seq analysis of sesame (Zhang et al., 2012) increased the number of known SSRs from 80 to several thousand with, on average, one genic-SSR per ~8 kb. Similarly, Haseneyer et al. (2011) sampled the transcriptomes of five winter rye inbred lines to identify 5,234 SNPs, which were then incorporated in a high-throughput SNP genotyping array, further demonstrating the value of RNA-seq as a tool for advanced molecular breeding.

Another striking example of the value of RNA-seq as an enabling technology is its application to advance the field of proteomics. High-throughput mass spectrometry-based protein identification relies on the availability of an extensive DNA sequence database in order to match experimentally determined peptide masses with the theoretical proteome generated by computationally translating transcripts. Indeed, the lack of extensive plant DNA sequence information and related resources is likely a contributing factor in the relatively slow progress in the arena of plant proteomics compared with proteome studies of other organisms for which high quality sequence has long been available. Lopez-Casado et al. (2012) recently demonstrated that RNA-seq-based transcriptome profiling can provide an effective data set for proteomic analysis of non-model organisms by *de novo* assembly of 454-based ESTs derived from the pollen of tomato (*Solanum lycopersicum*) and two wild relatives. Approximately the same number of proteins was identified when using either the RNA-seq-derived database, generated through a few 454 pyrosequencing runs, or a highly curated community database of tomato sequences generated over more than a decade. This suggests that RNA-seq will be invaluable in facilitating protein identification and that proteome studies need no longer be so taxonomically restricted.

## CHARACTERIZING TEMPORAL, SPATIAL, REGULATORY, AND EVOLUTIONARY TRANSCRIPTOME LANDSCAPES

As with previous large-scale transcript profiling platforms, including microarrays, RNA-seq is increasingly being adopted to examine transcriptional dynamics during various aspects of plant growth and development. For example, an analysis of the transcriptome of grape (*Vitis vinifera*) berries during three stages of development identified >6,500 genes that were expressed in a stage-specific manner (Zenoni et al., 2010). Evidence of even greater transcriptomic complexity was provided by the detection of 210 and 97 genes that undergo alternative spicing in one or two stages, respectively. Similarly, Wang et al. (2012) analyzed the transcriptome of radish (*Raphanus sativum*) roots at two developmental stages and found >21,000 genes to be differentially expressed, including genes strongly linking root development with starch and sucrose metabolism and with phenylpropanoid biosynthesis. The radish genome has yet to be sequenced, but comparative sequence analysis of the radish RNA-seq data and the *Brassica rapa *genome sequence lead to the discovery of 14,641 SSRs.

Most RNA-seq analyses target whole organs, or sets of organs, which inherently prevents the identification of cell or tissue type transcripts, and thus spatially coordinated structural and regulatory gene networks. Furthermore, transcripts that are expressed at extremely low levels, or that are specific to an uncommon cell type in a complex organ or tissue, may be diluted below the limit of detection. Accordingly, RNA-seq analysis of discrete tissues or cell types has the potential to both yield an important level of spatial information and substantially increase the depth of sequence coverage. As an example, Chen et al. (2010) detected more than 1,000 genes that are specifically or preferentially expressed in *Arabidopsis* male meiocytes that had been isolated by mechanically disrupting anthers with forceps and collecting the released meiocytes with a capillary pipette. However, acquiring tissue or cell-specific samples with any degree of precision and minimal contamination is often technically difficult, although several methods have been developed to facilitate this. For example, a cell type gene expression map of an *Arabidopsis* root was achieved by generating a set of transgenic *Arabidopsis* lines expressing green fluorescent protein (GFP) driven by various root cell type-specific promoters, digesting entire roots with cell wall degrading enzymes and fractionating the resulting protoplasts into distinct pools using an automated cell sorter (Birnbaum et al., 2003). The constituent root cell type-related transcriptomes were then analyzed using a microarray, providing a high resolution profile of the spatial variation in the root transcriptome. An alternative approach, which requires no prior genetic transformation or cell wall digestion, is laser capture microdissection (LCM), where a laser is used to excise and isolate samples from tissue sections with micron-scale resolution. This technique has been effectively used by plant researchers in conjunction with microarray analysis (Nakazono et al., 2003; Cai and Lashbrook, 2008; Agustï et al., 2009; Brooks et al., 2009; Matas et al., 2010). More recently, Matas et al. (2011) used LCM in combination with RNA-seq (454 pyrosequencing) analysis to profile the transcriptomes of the five principal tissues of the developing tomato fruit pericarp. Approximately 21,000 unigenes were identified, of which more than half showed ubiquitous expression, while other subsets showed clear cell type-specific expression patterns, providing insights into numerous aspects of fruit biology. A similar number of genes was identified in an LCM-based study of the ontogeny of maize (*Zea mays*) shoot apical meristems using RNA-seq coupled with Illumina-based NGS (Takacs et al., 2012). Interestingly, 59% of the transcripts were detected in all the samples, comprising the apical domains along a developmental gradient from maize embryos to seedlings; a value that is very similar to the percentage of unigenes present in all tissues of the tomato fruit (57%) reported by Matas et al. (2011), and the proportion of ubiquitously detected transcripts in the root cell sorting analysis (Birnbaum et al., 2003). RNA-seq profiling analyses of a number of mammalian tissues have also indicated a high proportion of ubiquitously expressed transcripts, which may indicate that this is a common feature of eukaryotes (Ramsköld et al., 2009).

In addition to studies focusing on transcriptional changes during development, RNA-seq has already shown itself to be a highly effective strategy to study plant responses and adaptations to abiotic and biotic stresses. For example, by analyzing RNA-seq data derived from sorghum (*Sorghum bicolor*) plants treated with abscisic acid (ABA) or polyethylene glycol, in conjunction with published transcriptome analysis for *Arabidopsis*, maize, and rice, Dugas et al. (2011) discovered >50 previously unknown drought-responsive genes. Similarly, RNA-seq was used to reveal massive changes in metabolism and cellular physiology of the green alga *Chlamydomonas reinhardtii* when the cells become deprived of sulfur, and to suggest molecular mechanisms that are used to tolerate sulfur deprivation (González-Ballester et al., 2010). Equivalent high resolution gene expression information has also resulted from studies of plant responses to pathogens and the complexities of the metabolic pathways associated with plant defense mechanisms. Published examples to date include a transcriptomic analysis of the infection of sorghum by the fungus *Bipolaris sorghicola* (Mizuno et al., 2012) and an investigation into the defense mechanisms of soybean that provide resistance to *Xanthomonas axonopodis*, by comparing resistant and susceptible near-isogenic lines (Kim et al., 2011).

As well as its applications to study spatial and temporal transcriptome dynamics, RNA-seq is also a potentially valuable tool to advance studies of plant evolution and polyploidy. As an illustration, a comparison of the leaf transcriptome of an allopolyploid relative of soybean with those of the two species that contributed to its homoelogous genome, allowed the determination of the contribution of the different genomes to the transcriptome (Ilut et al., 2012). Another study analyzed the transcriptome of nine distinct tissues of three species of the Poaceae family (Davidson et al., 2012) to determine whether orthologous genes from these three species exhibit the same expression patterns. Knowledge of parental imprinting has also been substantially advanced by deep transcriptome surveys. Despite the discovery of genetic imprinting in maize 40 years ago, only seven maize imprinted genes were reported before large-scale transcriptomic sequencing was applied to maize endosperm, leading to the discovery of 179 imprinted genes and 38 imprinted long ncRNAs (lncRNAs; Zhang et al., 2011). Studies of the embryo and endosperm of *Arabidopsis* and rice similarly increased the numbers of known imprinted genes and showed that imprinting is primarily endosperm-specific (Gehring et al., 2011; Hsieh et al., 2011; Luo et al., 2011).

We note that the studies cited in this section highlight the tremendous diversity of RNA-seq applications and the breadth of research fields in which it is being adopted, and the purpose is to provide examples, rather than a comprehensive list.

## IDENTIFYING AND CHARACTERIZING NOVEL NON-CODING RNAs

Small RNAs (sRNAs) play important roles in gene post-transcriptional regulation (Baulcombe, 2004; Zamore and Haley, 2005) and there is great interest in developing techniques to comprehensively profile sRNA populations. *In silico *analysis provides a rapid way to identify putative sRNA genes (Chen et al., 2003, 2011; Hirsch et al., 2006) but RNA-seq technology represents an excellent means for sRNA discovery and validation. Indeed, deep sequencing of sRNAs has already been extensively used to find new sRNAs and especially microRNAs (miRNAs; Lu et al., 2005; Moxon et al., 2008; Szittya et al., 2008; Pantaleo et al., 2010; Song et al., 2010; Ferreira et al., 2012, Xia et al., 2012).

Characterization of miRNAs regulatory functions is likely to be facilitated by determining tissue-specific expression pattern, as shown by Breakfield et al. (2012) where RNA-seq was used to identify sRNAs from five *Arabidopsis* root tissues. Some sRNAs were expressed in all five tissues while others were tissue-specific, and some fluctuations in miRNA expression were also observed across developmental zones. In addition, growing numbers of RNA-seq studies are revealing the spatial and temporal differential expression of sRNAs in plant organs (Hirsch et al., 2006; Moxon et al., 2008; Pantaleo et al., 2010; Calviño et al., 2011). The availability of high-throughput RNA-seq data allowed Yang et al. (2012) to mine these databases and discover that ~12% of 354 high-confidence miRNA binding sites identified in *Arabidopsis* are affected by alternative splicing. The frequency of alternative slicing at miRNA binding sites is significantly higher than that at other regions, suggesting that alternative splicing is a significant regulatory mechanism. Small ncRNAs (sncRNAs) are also implicated in abiotic stresses and many miRNAs and other sRNAs have been shown to be differentially expressed under phosphate starvation in *Arabidopsis* roots and shoots (Hsieh et al., 2009), or under cold conditions (Zhang et al., 2009). The large amounts of data easily generated by RNA-seq also enable comparisons of sRNA populations between species, as demonstrated by Moxon et al. (2008), who found two tomato miRNAs that were previously believed to be specific to *Arabidopsis* or moss. In contrast to the numerous studies of plant sRNAs, far less is known about lncRNAs (>200 nt), especially in plants, and few plant lncRNAs have been characterized to date (Au et al., 2011; Kim and Sung, 2011; Zhu and Wang, 2012). Those that have been identified did not involve RNA-seq and so this represents an area with great potential for discovery.

Finally, sRNAs have been recently characterized in the context of association with epigenome modifications, including cytosine methylation of genomic DNA. While the majority of such work has involved animal systems, whole genome methylation analysis of epigenetic variation in *Arabidopsis* and rice embryo development, combined with sRNA analysis of the same tissues, confirmed a link between demethylation of certain gene promoters and associated miniature inverted repeats with changes in sRNA abundance (Cokus et al., 2008; Lister et al., 2008; Zemach et al., 2010). Interestingly, while promoter demethylation of tomato ripening genes was also recently described, it did not occur in conjunction with notable changes in sRNAs (Zhong et al., 2013). Genome-scale analyses of gene and sRNA expression via RNA-seq, combined with whole genome methylation analyses are now facilitating the exploration of epigenomes in ways that could not have been considered prior to these high-throughput sequencing technologies.

## FROM CO-EXPRESSION NETWORKS TO INTEGRATIVE DATA ANALYSIS

Sequencing whole transcriptomes provides a high degree of detail, but deriving useful biological information from a long list of expressed genes is typically not trivial. One approach to using such information to develop and refine hypotheses is to construct networks of co-expressed genes and to use gene ontology (GO) information to help highlight important gene candidates as critical components of functional networks. Many such “guilt-by-association” gene co-expression networks have been constructed based on microarray data (Manfield et al., 2006; Mao et al., 2009; Childs et al., 2011; Tohge and Fernie, 2012) and are now being more widely adopted to evaluate RNA-seq data (Dugas et al., 2011; Iancu et al., 2012; Li et al., 2012). Indeed, the broad dynamic range of transcript level detection allowed by RNA-seq profiling, and particularly the detection of low-abundance transcripts, facilitates meaningful discrimination between different strengths of association in correlation analyses (Iancu et al., 2012). The correlations between different genes forming the expression network are therefore more robust and the overall expression network quality is generally superior to that generated using microarrays.

Gene ontology enrichment analysis of RNA-seq data often illustrates the complexity of interacting pathways. For example, in a study of abiotic stress responses in maize, transcripts associated with numerous GO classifications were affected by drought treatment, including the categories “carbohydrate metabolic process,” “response to oxidative stress,” and “cell division,” among others (Kakumanu et al., 2012). The authors also showed that variations in GO term representation between organs can also provide valuable information and specifically, the drought-treated fertilized maize ovary exhibits a massive decrease of mRNAs involved in cell division and cell cycle, which could be the direct cause of the previously observed embryo abortion under drought conditions.

Functional networks can be made more robust by integrating multiple data types and various studies have coupled RNA-seq with proteomics and/or metabolomics, characterizing the apparent downstream consequences of transcript level variation. An example of such a “systems” study involved a comparative analysis of the transcriptome, proteome, and targeted metabolome of soybean seeds from transgenic lines with suppressed expression of the storage proteins glycinin and conglycinin (Schmidt et al., 2011). This study showed no direct correlation between the levels of transcripts, proteins, and metabolites. Conversely, a significant correlation was found between the high expression of fatty acid synthesis genes and the high oil content in oil palm mesocarp (Bourgis et al., 2011). These studies further demonstrate the value of characterizing biological processes from multiple “omics” perspectives, each of which can provide insights into different regulatory mechanisms. Surveying the metabolome and transcriptome in parallel can also help identify candidate genes involved in complex metabolic pathways. For example, Desgagné-Penix et al. (2012) took advantage of several opium poppy (*Papaver somniferum*) cultivars with known differential levels of benzylisoquinoline alkaloids (BIAs) and used a combination of RNA-seq and mass spectrometry to pinpoint key regulatory steps of the almost completely defined morphine biosynthetic pathway, leading to the discovery of candidate genes implicated in BIA metabolism.

These examples show that the integration of transcriptomics, proteomics, and metabolomics can expose complex biological and biochemical interactions, paving the way to elucidate relationships between genotype and phenotype. Even greater resolution can be achieved by targeting tissues instead of whole organs (Rogers et al., 2012).

## A GROWING PORTFOLIO OF RNA-seq ANALYTICAL STRATEGIES

RNA-seq technologies can be adapted to answer-specific biological questions. Four different adaptations or applications are described here.

### STRAND-SPECIFIC RNA-seq

**FIGURE 1:**
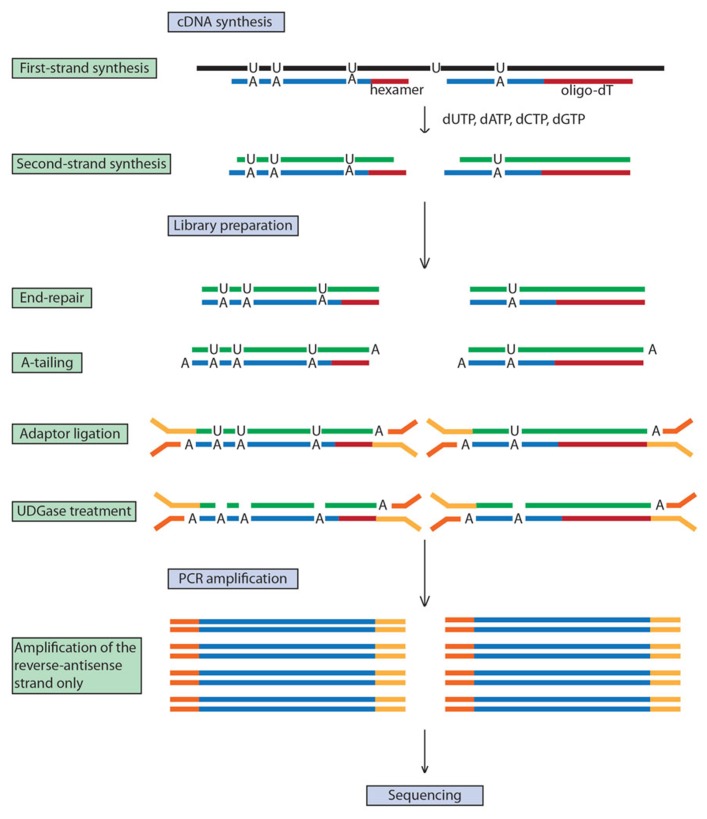
**dUTP-based strand-specific RNA-seq.** Strand-specificity is achieved by the second-strand cDNA incorporating dUTPs instead of dTTPs. Digestion of dUTPs by uracil-DNA glycosylase (UGDase) prevents this strand from being PCR amplified, conferring single-strand specificity.

Standard RNA-seq methods do not discriminate between the DNA strands on which the RNAs are encoded. However, the ability to map a transcript to its specific coding strand is desirable as it improves transcript mapping accuracy by identifying non-coding antisense transcripts that may be involved in regulation at the messenger or at the chromatin levels (Ponting et al., 2009; Liu et al., 2010), helps determine the relative expression level of two genes on opposite DNA strands as well as their exact length, and allows the identification of the transcribed strand of ncRNAs. Levin et al. (2010) compared seven library construction methods to enable strand-specific RNA-seq analysis and overall, a dUTP method (Parkhomchuk et al., 2009) was the most accurate and has the advantage of being compatible with paired-end sequencing. This method has been successfully applied to plant RNA-seq with adaptations rendering it low-cost and high-throughput (Wang et al., 2011; Zhong et al., 2011). In short, the first cDNA strand is synthesized with dNTP while dUTP is incorporated in the second cDNA strand. After end repair, A-tailing and adaptor ligation, the dUTP-containing strand is digested and the remaining strand is PCR-amplified conferring strand specificity (**Figure 1**). As an example of the value of strand information, a study of tomato gene expression showed that while the majority of genes in the tissues analyzed had effectively the same expression profiles when analyzed by either double-stranded or strand-specific RNA-seq, approximately 5% of transcripts were associated with misleading results when assayed by double-stranded RNA-seq (dsRNA-seq) alone (Zhong et al., 2011).

### BULKED SEGREGANT RNA-seq

**FIGURE 2:**
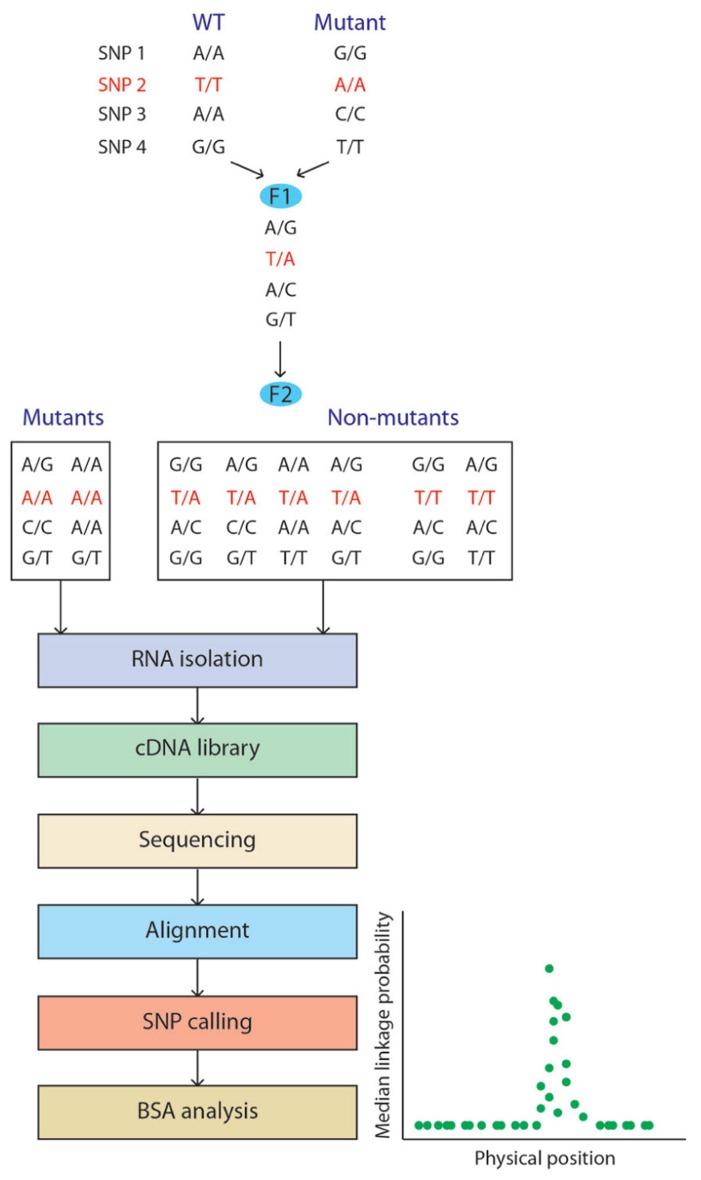
**Bulked segregant analysis (BSA) using RNA-seq.** Wild-type and mutant plants from different genetic backgrounds are crossed. In this example, four SNPs are listed with SNP 2 being closely related to the mutation to map. A plant from the F1 generation is selfed to generate the F2 segregating population. Mutant and non-mutant plants are processed independently and the BSA analysis allows visualization of the probability of each SNP marker being in complete linkage disequilibrium with the mutated gene.

Liu et al. (2012) demonstrated the application of RNA-seq for bulked segregant analysis (BSA) by mapping the maize mutant gene *gl3*. Transcriptome profiling is applied to a pool of two samples generated by mixing a bulk of mutant and wild-type (WT) plants (**Figure 2**). The mapping of the mutated gene is based on genetic linkage where linkage disequilibrium between markers and the causal gene is determined by quantifying the allelic frequencies between the two samples, giving the map position of the gene responsible for the mutant phenotype. Fine mapping of the mutated gene is facilitated by the RNA-seq data as its expression will often be down-regulated compared to the WT pool. Additionally, the SNPs linked to the mutated gene can be used for chromosome walking. Using RNA-seq for this purpose has therefore numerous advantages: (i) having a reference genome is not a prerequisite as *de novo* assembly of the transcriptome based on the RNA-seq data is sufficient; (ii) markers can be generated from the experimental data; and (iii) differential expression profiles between the mutant and the WT are generated at no extra cost. Furthermore, this approach can be modified to perform genome-wide association (GWAS) studies, accelerating breeding initiatives by providing markers targeting both genetic sequence (e.g., SNPs) and gene expression, using them to identify the genomic regions associated with the traits of interest (Harper et al., 2012).

### DOUBLE-STRANDED RNA-seq

**FIGURE 3:**
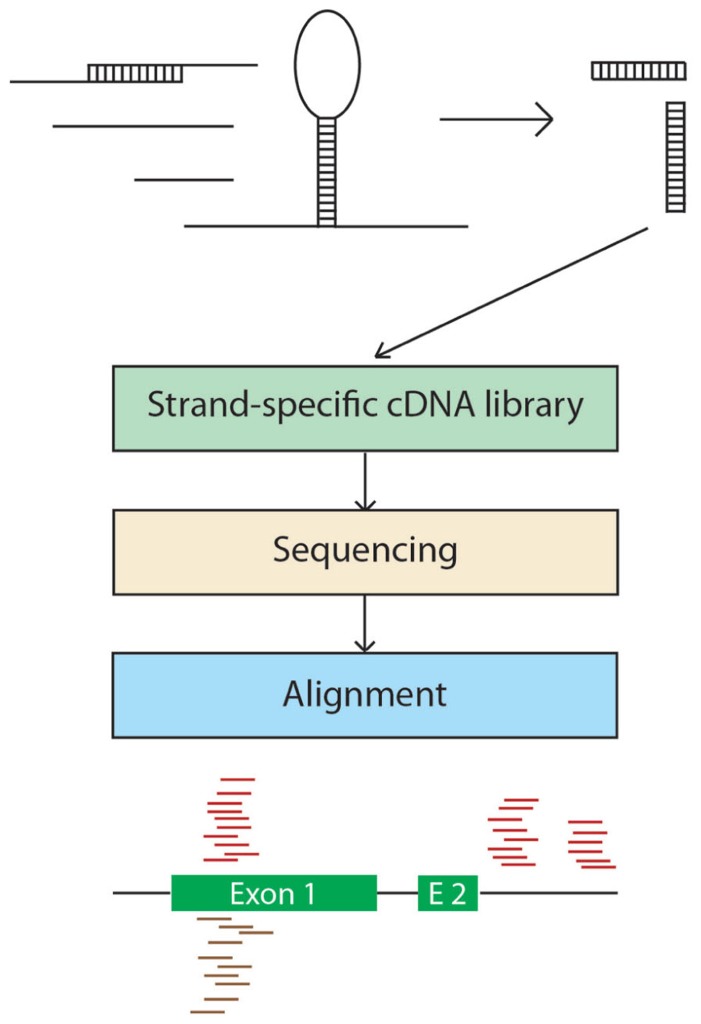
**Double-stranded RNA-seq.** A total RNA sample is isolated and single-stranded RNA is digested with a single-strand-specific ribonuclease. The reads generated from the strand-specific cDNA library are aligned to the genomic sequence and intra or inter-molecular pairing can be inferred based on the strand specificity of the mapped reads.

Secondary structures of RNAs are central to their function, maturation, and regulation; however, little is known about the double-stranded features of most RNAs. Zheng et al. (2010) reported an experimental strategy to survey RNA secondary structures in an analysis of the double-stranded species of RNAs from *Arabidopsis* flower buds. Specifically, the authors sequenced only the double-stranded RNAs (dsRNAs) and the double-stranded segments of RNAs by digesting the single-stranded RNAs with a ribominus treatment prior to library construction (**Figure 3**). As expected, highly structured RNA classes (e.g., rRNA, tRNA, and snRNA) were highly represented in the reads but, interestingly, other regions of various mRNAs, including introns, exons, and 5′ and 3′ UTRs were also present, indicating the presence of mRNA secondary structures. Moreover, the double-stranded regions of the introns, 3′ and 5′ UTRs appeared to be conserved, suggesting a common function. Notably, certain regions of the genome appear to be responsible for producing more dsRNAs than others, with transposable elements representing nearly 60% of these “hotspots.”

### DIFFERENTIAL RNA-seq

**FIGURE 4:**
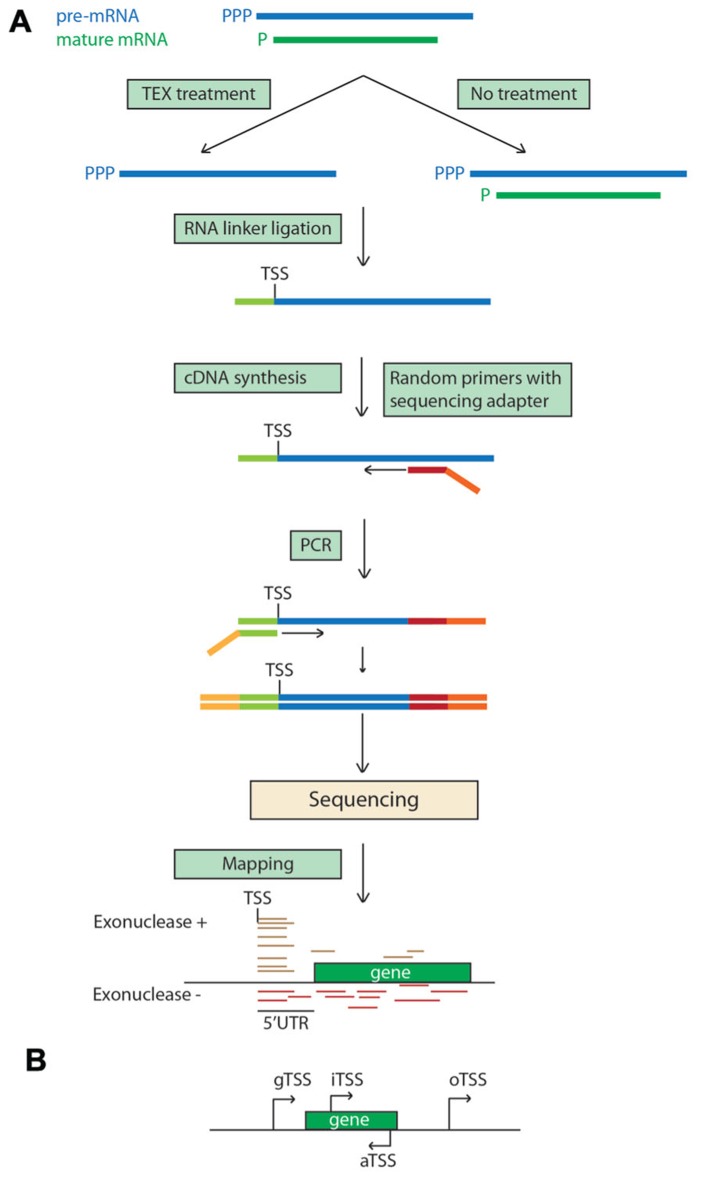
**Differential RNA-seq.** **(A)** 5′ monophosphorylated chloroplastic mature transcripts are degraded with Terminator 5′-phosphate-dependent exonuclease (TEX) in the TEX treatment, enriching the sample with pre-mRNAs. The 5′ ends of the pre-mRNAs are enriched in the treated sample because they are protected by the triphosphate and the TEX treatment removes degraded mRNAs (Sharma et al., 2010). The enrichment can be enhanced by shearing the RNA before the TEX treatment. Sequencing reads starting exactly at the same nucleotide position in the exonuclease treatment denote the transcription start site. **(B)** Example of localization of different TSS. TSS, transcription start site; gTSS, gene TSS; iTSS, internal TSS; aTSS, antisense TSS; oTSS, orphan TSS.

Differential RNA-seq (dRNA-seq) is based on a comparison of a terminator exonuclease treated RNA sample with its non-treated counterpart (**Figure 4**). The treatment removes the processed transcripts by degrading 5′ monophosphate RNAs, which are characteristic of prokaryotic RNAs, and the primary unprocessed transcripts are not affected due to the presence of a 5′ triphosphate. By comparing the maps of the reads derived from each sample, TSSs of operons are identified. dRNA-seq was first used to examine the transcriptome of the human pathogen *Helicobacter pylori* (Sharma et al., 2010) and subsequently in studies of various prokaryotes, including the plant pathogen *Pseudomonas syringae* (Filiatrault et al., 2011). This method was used to map TSSs of barley chloroplastic RNAs (Zhelyazkova et al., 2012) and was possible as they have the same 5′ monophosphate structure as prokaryotic RNAs, reflecting the endosymbiotic origin of chloroplasts. Four categories of TSSs were identified in this study: gTSSs (g: gene) located within 750 nucleotides upstream of annotated genes (the majority of TSSs); iTSSs (i: internal) located within annotated genes and giving rise to sense transcripts; aTSSs (a: antisense) giving rise to antisense transcripts; and oTSSs (o: orphan) located in intergenic regions. The analysis revealed that some individual transcriptional units of the chloroplastic operons can be transcribed individually as suggested by iTSSs and that ~35% of chloroplastic genes have aTSSs or oTSSs, providing evidence of extensive ncRNAs synthesis in chloroplasts.

## CONCLUDING REMARKS

RNA-sequencing is now well-established as a versatile platform with applications in an ever growing number of fields of plant biology research. Ongoing developments in sequencing technologies, such as increased read lengths, greater numbers of reads per run, and advanced computational tools to facilitate sequence assembly, analysis, and integration with orthogonal data sets will further accelerate the breadth and frequency of its adoption by plant scientists. An important issue that still needs to be addressed is the inherent bias introduced by the different steps of library construction and so the tantalizing prospect of direct RNA-seq (Ozsolak and Milos, 2011) has great promise in this regard.

## Conflict of Interest Statement

The authors declare that the research was conducted in the absence of any commercial or financial relationships that could be construed as a potential conflict of interest.

## Footnotes

1 http://genomevolution.org/wiki/index.php/Sequenced_plant_genomes

2 http://www.onekp.com
